# Caffeine-clozapine interaction associated with severe toxicity and multiorgan system failure: a case report

**DOI:** 10.1186/s12888-021-03199-x

**Published:** 2021-04-13

**Authors:** Alex Yartsev, Carmelle Peisah

**Affiliations:** 1 Westmead Hospital Intensive Care Unit, Sydney, Australia; 2 University New South Wales, Sydney, Australia

**Keywords:** Clozapine, Caffeine, Interaction, Multiorgan system failure, Case report

## Abstract

**Background:**

Caffeine is a known inhibitor of Clozapine metabolism mediated by inhibition of CYP1A2. Hitherto, the effects of caffeine on Clozapine levels have always been modest, as have the clinical manifestations of toxicity resulting from their interaction. We present a case of severe toxicity associated with the co-consumption of caffeine and Clozapine culminating in life-threatening complications requiring management in Intensive Care.

**Case presentation:**

A 34 year old male with a history of chronic schizophrenia, who had been managed stably on 400 mg Clozapine for the previous 5 years, changed his dietary behaviour and began consuming caffeine-containing energy drinks over the course of 3 weeks. The total daily dose of caffeine was estimated as 600 mg/day (four cans of Red Bull). He subsequently presented to the Emergency Department with life-threatening Clozapine toxicity, resulting in a decreased level of consciousness, severe metabolic acidosis, acute respiratory failure, raised inflammatory markers and acute renal failure attributed to interstitial nephritis. Maximum recorded Clozapine level was 1796 ng/ml.

**Conclusions:**

This case describes the interaction between a common caffeine-containing beverage and a commonly prescribed antipsychotic medication, associated with severe adverse effects. We call for clinical and scientific attention to the possible interaction between these substances and draw attention to the implications for prescribing practices and patient counselling.

## Background

Clozapine is a commonly prescribed medication indicated for the management of treatment-resistant schizophrenia, with a worldwide pattern of increasing use [1–3]. Clozapine is a dibenzazepine derivative which is metabolised predominantly by the liver, the two principal metabolites being desmethylClozapine and Clozapine N-oxide. The demethylation of Clozapine which produces desmethylClozapine is performed by CYP1A2 [4]. Caffeine inhibits the metabolism of Clozapine, probably by inhibition of CYP1A2. The effect of caffeine on Clozapine levels in studies of healthy volunteers has always been modest. For example, serum Clozapine levels increased by only 19% following co-administration of Clozapine (12.5 mg) and caffeine (100 mg) in healthy young adults [5, 6]. Further, the clinical manifestations of toxicity resulting from the interaction of Clozapine and caffeine hitherto described have been similarly relatively mild and have included stiffness, abrupt onset of arousal and exacerbation of psychosis, all of which were easily reversible [6, 7]. However, significant interindividual variability exists in pharmacokinetics of commonly used antipsychotics^8^. The aforementioned case studies report a Clozapine dose of 200 mg and a caffeine dose of 425-850 mg/day in one scenario [7] and a Clozapine dose of 550 mg with a caffeine intake of 200 mg/d the other [6]. We are unaware of any previous reports of severe, life-threatening interactions between caffeine and Clozapine. We present a case of a patient with chronic schizophrenia managed with a stable long-term dose of Clozapine who presented with severe Clozapine toxicity culminating in multisystem organ failure due to a change in caffeine intake. While this severe adverse interaction is unusual, consumption of high energy drinks is part of a raft of poor nutritional habits often associated with serious mental illness such as schizophrenia both in a chronic state [8–10], as well as a manifestation of relapse [11, 12]. Hence, the importance of shedding light on this case.

## Case presentation

A 35 year-old male was brought to the emergency department by ambulance after being found unconscious in his home.

### History of presenting complaint

He was last seen to be alert twenty-four hours prior. On the day of his presentation, his carer found him sitting slumped against the wall on the floor, obtunded and tachypnoeic. The ambulance officers made a note of “energy drinks spread over living room table” in his home. At triage in the emergency department, his respiratory rate was 50, heart rate 110, non-invasive systolic blood pressure 129 mmHg. Glasgow coma score (GCS) was initially scored as 14 (E = 4 V = 4 M = 6) and the blood sugar level (BSL) was 32.5 mmol/L. A tympanic temperature of 38.0 C was recorded. Arterial blood gas analysis revealed a profound metabolic acidosis with pH of 6.91, PaCO_2_ 44 mmHg, HCO_3_^−^ 10, lactate of 6.2, and an anion gap of 26 mmol/L. Point-of-care testing revealed a serum ketone level of 3.0. Given the raised BSL and decreased level of consciousness, a provisional diagnosis of hyperosmolar hyperglycaemic syndrome with diabetic ketoacidosis was made. Shortly after triage, the patient’s level of consciousness deteriorated, with a GCS score of 6 (E = 1 V = 1 M = 4). He was intubated and transferred to the Intensive Care Unit (ICU). A urine drug screen was obtained prior to the intubation, and was negative for all tested substances (amphetamine, cannabis, cocaine, MDMA, benzodiazepines and opiates).

### Progress during ICU admission

Over the subsequent 2 days of his stay in the ICU, the patient remained unconscious with minimal sedation, and dependent on mechanical ventilation. He required modest ventilator support and his fraction of inspired oxygen (FiO_2_) was weaned to 0.3 within the first twenty-four hours.

He remained stable haemodynamically, and did not require any vasoactive agents to support his blood pressure. During the first 2 days of ICU admission he remained febrile and a significant inflammatory marker rise was observed, with a CRP of 520 mg/L and a procalcitonin of 13.21 μg/L, rising at maximum over his course of stay to 40.77 μg/L. As septic shock was suspected, empiric antimicrobial therapy with meropenem (1 g every 8 h) was commenced. He underwent a computed tomography scan (CT) of the head, chest, abdomen and pelvis, which did not reveal any foci of infection. Specifically, no structural abnormality of the kidneys was detected, nor any changes in the lungs which might have been associated with aspiration pneumonia. Blood cultures and urine cultures did not yield any pathogenic organisms, and COVID-19 PCR was negative. Meropenem was ceased on the third day of admission, as there remained no clinical features of infection.

As the patient had become anuric and was developing clinically significant fluid overload, a vas cath was placed and continuous renal replacement therapy was commenced (continuous veno-venous haemodiafiltration, CVVHDF). As the serum Clozapine level taken on admission to the emergency department had now became available (1796 ng/ml), Clozapine-induced interstitial nephritis was raised as a possibility, and a trial of intravenous methylprednisolone was commenced empirically (as the patient’s morbidly obese body habitus did not lend itself to a safe renal biopsy). Methylprednisolone (250 mg daily) was used for a total duration of 5 days.

After day three of ICU admission, the patient had regained consciousness, and was able to obey simple commands. Having been commenced on olanzapine earlier in the admission for agitation, this was weaned and ceased and aripiprazole (5 mg daily) commenced via NGT (ie prior to extubation) chosen for its safest use in renal failure and in hyperglycaemia. This was well-tolerated and continued for the remainder of his admission.

**Fig. 1:**
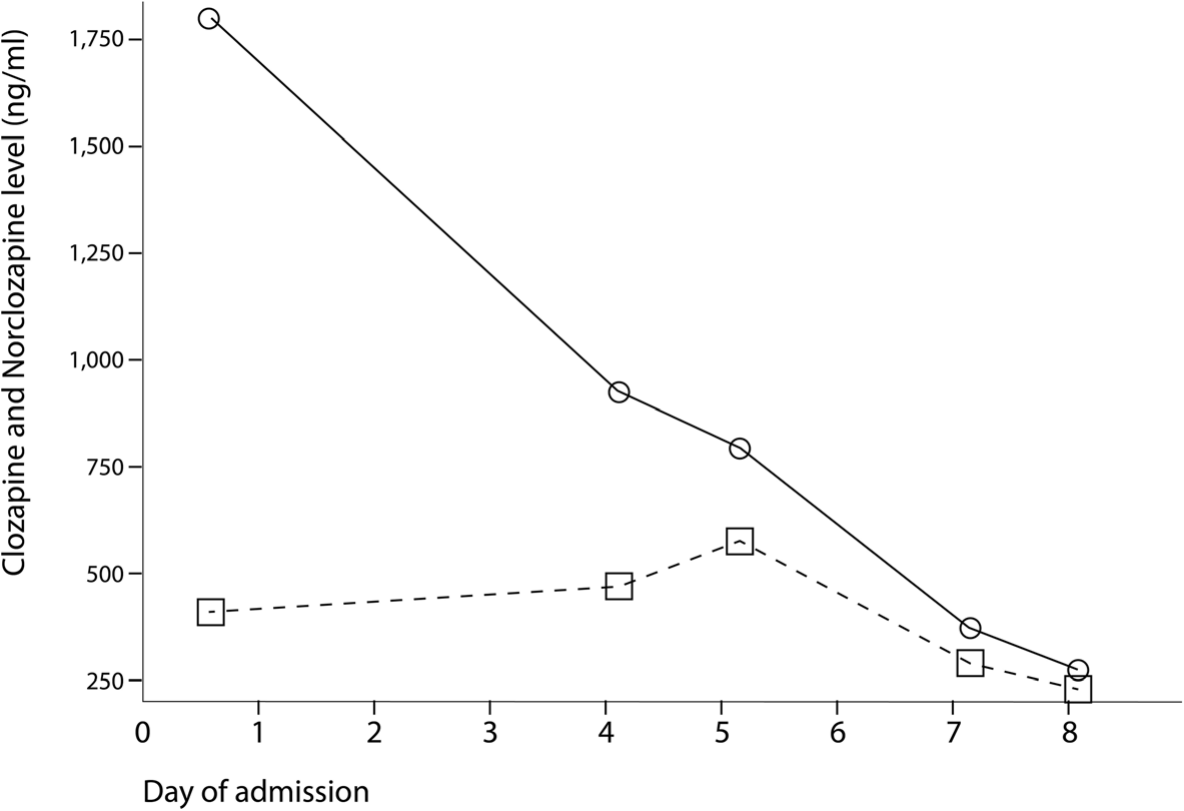
“Clozapine level and NorClozapine level”. Solid line: Clozapine level. Dashed line: norClozapine level

After fluid removal by haemofiltration the patient was extubated on Day 4 of ICU admission, with no further respiratory sequelae. At this stage, the Clozapine level had decreased to 926 μg/L. (see Fig. 1).

### Past medical and surgical history

The patient had a background history of morbid obesity (weight = 142 kg; BMI 48.9 kg/m^2^), childhood asthma, gastro-oesophageal reflux disease, hypercholesterolaemia and impaired glucose tolerance. His regular medications consisted of Clozapine 400 mg nocte, esomeprazole 20 mg mane, atorvastatin 10 mg nocte, and metformin 500 mg nocte. He was a lifelong non-smoker, and consumed alcohol every day (4 beers, or approximately 60 g of ethanol per day) until the week prior to presentation (see below).

### Past psychiatric history

The patient was commenced on Clozapine (400 mg nocte) 2 years prior to these events, following a prolonged inpatient admission to a mental health facility during which several other agents were trialled unsuccessfully. His psychiatrist confirmed that on Clozapine the patient had remained stable with no relapses and regular clinic attendance. Clozapine levels in the community had been within the normal therapeutic range.

Prior to presentation he had been living alone and working as a cleaner up until the COVID pandemic. He was relatively independent in activities of daily living, although received assistance weekly with grocery shopping from a support worker. He self- administered medications and Clozapine levels had been stable during outpatient therapy, as had been his haematological indices on regular screening, with the exception of mildly reduced lymphocytes at times (with preservation of neutrophil levels). These historical data suggest that this patient was not suffering from any cardiovascular or immunological complications of Clozapine therapy which might otherwise explain his presentation and multi-organ system failure.

### Mental status examination

Upon review of his mental status following extubation, the patient was appropriate with euthymic mood and bright, reactive affect. There was no evidence of active psychotic symptoms (i.e. no thought disorder, no perceptual abnormalities nor abnormalities of content) or delirium. He denied any suicidal ideation either cross-sectionally or prior to presentation, and rather reported usual compliance with his medication in the weeks leading up to his presentation. He demonstrated good insight and judgement, including help seeking and wanting to remain on psychotropics. According to his own account, during the week before admission, he had decreased his intake of alcohol to zero, while increasing his intake of energy drinks to achieve weight loss with his daily consumption of caffeine fluctuating between 150 and 450 mg/day (between two and six 250 ml cans of Red Bull per day, with a caffeine concentration of 30 mg/100 ml).

### Progress and outcome

The patient was discharged from the ICU after ten days. Methylprednisolone had transitioned to oral prednisolone, the dose of which was weaned gradually over 3 weeks, and ceased. Other complications of his stay included a left lower limb DVT and HITS, which had required intravenous bivalirudin as a bridge to warfarin therapy. His renal function remained impaired following discharge from the ICU, and renormalised only after fourteen days. He was discharged home after a total hospital stay of twenty-seven days. On consultation with the patient and his regular psychiatrist, aripiprazole was continued instead of Clozapine as his regular therapy.

## Discussion and conclusions

This case describes significant morbidity associated with Clozapine toxicity, which most likely resulted from the interaction between Clozapine and caffeine in an otherwise stable patient with chronic schizophrenia.

Clozapine is a known cause of acute interstitial nephritis [13], which manifests as acute kidney injury characterised by the histological finding of inflammatory oedema in the tubulointerstitium of the kidney. Although it is usually described in association with fever, skin rash, and eosinophilia, in reality only a minority of patients demonstrate these “classic” features [14]. Case reports of Clozapine-induced acute interstitial nephritis generally describe renal impairment of subacute onset in stable patients undergoing outpatient treatment [13]. Only one case of Clozapine-induced nephritis required intermittent haemodialysis [15].

Clozapine use per se is also a known cause of elevated inflammatory markers and fever, although this phenomenon is usually associated with Clozapine-induced myocarditis. The presence of CRP elevation in association with Clozapine therapy and without myocarditis is also known from case reports [16, 17] where a modest CRP elevation was observed (55.4–122 mg/L). However, in this case, the patient remained febrile for forty-eight hours with temperatures exceeding 39.0 C and had a CRP value of 520 mg/L, more consistent with sepsis. Similarly, a modestly elevated procalcitonin has been reported in association with Clozapine use [18] although in this case, procalcitonin levels were in a range usually associated with severe septic shock. Nothwithstanding this, the only positive peripheral blood culture was collected at the time of his admission to the ED, and had grown *S.epidermitis*, which was dismissed as a contaminant.

The other manifestation of severe Clozapine toxicity illustrated here was hyperosmolar hyperglycaemic syndrome with diabetic ketoacidosis, to which the patient was likely prone given his pre-existing metabolic syndrome and Clozapine use [19, 20]. These pre-existing conditions distinguish patients with schizophrenia on long-term antipsychotics such as Clozapine from healthy young adults – upon whom some of the drug interaction studies have been based – and go towards explaining the severity of the toxicity seen in this case.

The risk of drug interaction – not only involving therapeutic drugs but also recreational agents such as caffeine - must be taken into account when prescribing antipsychotic agents to patients with schizophrenia. This is all the more so because the consumption of caffeinated beverages- whether it be in the form of energy drinks, diet drinks or coffee - is almost ubiquitous and often perceived as harmless by patients. Moreover, the desire to lose weight – the driver in this case - is a natural and desirable response to the weight gain associated with antipsychotic drug use. However, the question remains: how much is “significant amounts of caffeine?” For example, Ellison and Dufresne suggested that caffeine can appreciably inhibit CYP1A2 with the equivalent of three cups of coffee per day [21], or approximately 3 mg/kg of caffeine. More conservatively, de Leon referred to “dramatic changes in caffeine intake” by more than one cup of coffee or two cans of caffeinated soda [22]. By removing caffeine (on average 162 mg/day) from the diet of patients who were receiving Clozapine monotherapy (271 +/− 102 mg/day) and neither alcohol nor any other medication, Carillo et al. [23] were able to reduce Clozapine levels by 46% (from 486 to 306 ng/mL). Given the interindividual variability in pharmacokinetics [24] there is no definitive answer, other than to arm the patient with the information and risks.

This case of severe Clozapine toxicity may not be attributed entirely to the interaction between Clozapine and caffeine. Clozapine is a substrate for multiple CYP450 enzymes, which include CYP2D6 and CYP3A4 as well as the CYP1A2 pathway affected by caffeine [4]. The patient was also regularly prescribed atorvastatin (10 mg nocte) which is known to inhibit CYP3A4 [25], esomeprazole (20 mg mane) which is not known to inhibit CYP450 enzymes [26] but which may compete with Clozapine as a substrate for CYP3A4, and metformin which does not undergo hepatic metabolism. The doses of these regular medications had remained unchanged for at least 12 months prior to this presentation, during which time the patient had stable Clozapine levels, which makes it unlikely that an atorvastatin-Clozapine interaction had contributed significantly to this sudden onset of severe toxicity. Esomeprazole, by acting as a competitive substrate for CYP3A4, may have contributed to Clozapine toxicity by eliminating this alternative pathway of metabolism, when clearance by the CYP1A2 pathway became unavailable. The patient had also been drinking up to 4 standard drinks of alcohol per day up util 1 week prior to his presentation. Ethyl alcohol is a known inducer of CYP3A4 [27], and the withdrawal of this effect (resulting in reduced CYP3A4 activity) could have contributed to decreased Clozapine clearance by increasing the reliance on the CYP1A2 pathway, which is inhibited by caffeine.

Patient education regarding the potential for drug interaction with specific enquiry into caffeine consumption should form a routine part of psychiatric clinical practice. The severity of the manifestations of Clozapine toxicity resulting from co-consumption of significant amounts of caffeine behoves us to warn our patients of this potential for serious harm from these interactions. Caffeine is not worth dying for.

## Data Availability

Patient data supporting the results reported in the article can be obtained from the corresponding author by email.

## Abbreviations

ICU: Intensive Care Unit
GCS: Glasgow Coma Scale
BSL: Blood Sugar Level
SBE: Standard Base Excess
CVVHDF: Continuous Veno-Venous HaemoDiaFiltration
NGT: NasoGastric Tube
HITS: Heparin-Induced Thrombocytopenia Syndrome
DVT: Deep Venous Thrombosis
